# The Safety of the Neighborhood Environment and Physical Activity in Czech and Polish Adolescents

**DOI:** 10.3390/ijerph15010126

**Published:** 2018-01-12

**Authors:** Josef Mitáš, Krzysztof Sas-Nowosielski, Dorota Groffik, Karel Frömel

**Affiliations:** 1 Institute of Active Lifestyle, Faculty of Physical Culture, Palacký University, 77111 Olomouc, Czech Republic; karel.fromel@upol.cz; 2 Department of Physical Education, Academy of Physical Education, 40065 Katowice, Poland; k.sas-nowosielski@awf.katowice.pl (K.S.-N.); d.groffik@awf.katowice.pl (D.G.)

**Keywords:** built environment, IPAQ-long form, recommendation, steps

## Abstract

(1) *Background*: An increase in or at least the sustainment of walking activities across a wide section of the population is a crucial health-related task for Central and East European countries. The aim of this study was to assess the associations between adolescents’ walking activities and various levels of perceived safety of the built environment in differing socio-demographic backgrounds of Poland and the Czech Republic. Furthermore, we aimed to determine major moderators affecting the walking habits of adolescents in areas with different levels of walkability. (2) *Methods*: The surveys were conducted during the 2008–2009 and 2013–2014 school years in 24 Polish and 35 Czech secondary schools, with a sample of 2001 adolescents. All participants completed the International Physical Activity Questionnaire–Long Form and the NEWS–Abbreviated. Selected students took part in objective weekly monitoring of physical activity (PA). (3) *Results*: Boys and girls who perceived their neighborhood environment as the safest were significantly more likely to meet the recommendations for leisure-time walking. Adolescents from the safest environment achieved 11,024 steps/day on average, while those from the least safe environment achieved 9686 steps/day. (4) *Conclusions*: A safe neighborhood environment significantly predicts walking activities among girls. Environmental safety improvement can support the active transport and better use of leisure time PA.

## 1. Introduction

Physical activity (PA) has been shown to be an effective preventive factor for the prevalence of type 2 diabetes that has rapidly increased in recent years among adolescents and is a causative factor for preventable deaths [1]. However, 81% of adolescents aged 11–17 years were estimated to be insufficiently physically active in 2010, globally [2], which might be influenced by neighborhood safety [3]. Less physical activity attributable to a perception of the neighborhood environment as being unsafe because of road traffic, stray dogs, and so on has been well documented, especially in adults. The environmental variables are ubiquitous and can have wide effects on the population; however, this makes them very difficult to study [4]. A review on correlates of adult PA reported a significant positive association between perceived neighborhood safety and physical activity [4]. In contrary adults, adolescents’ perception of neighborhood safety concerns was not correlated with physical activity [5], which might be limited due to a lack of evidence in this age group. One of the studies on youth living in diverse neighborhoods indicated that levels of youth recreational PA vary by neighborhood of residence, partially influenced by neighborhood-level socioeconomic characteristics [6]. Perceived neighborhood safety may serve as a barrier to physical activity in low-income setting neighborhoods [7]. The most robust correlates for children were walkability, traffic speed and volume, land-use mix (proximity of homes and destinations), and access or proximity to recreation facilities [8,9]. Other studies have indicated that the level of PA might be influenced by relatively higher levels of non-motorized and transit trip-making among adolescents without driver’s licenses [10,11], but no significant correlations were found between environmental variables and body mass index (BMI) percentile for girls or boys [11]. In a recent national study on the built environment and PA, these factors were not limiting for the total level of weekly PA in Czech adults [12]. No other Central European studies have monitored these associations either in adults or adolescents. Developing countries often look for a model of successful socio-demographic growth in more developed countries, despite the negative consequences of some patterns of expansion [13]. The research findings in the Czech Republic, however, indicate that safety of the neighborhood environments does not significantly affect the total weekly PA and walking in adults [12]. This trend should also be studied in similar environments in other Central European countries, where no evidence has yet been found.

As existing studies have not simultaneously considered the impact of all built environment features, it is difficult to determine features that most strongly affect the PA levels of adolescents [14]. Development of the Central European countries also includes a change in the perception of neighborhood security, especially with regard to repeating the creation of newly built obesogenic infrastructure. The aim of building a healthy environment should be influenced by factors such as car development, safe public transportation, the creation of walkable communities, and support for safe active transportation to schools. This is especially important as children are more active outdoors than indoors [15]. However, between 2001 and 2011, the active transport of Czech adolescents to schools decreased significantly [16,17]. Unfortunately, there is no reported trend study related to Polish youths’ travel behavior. In polish adults, it was found that, between 2003 and 2014, the total level PA as well as the level of commuting PA significantly decreased [18]. There is only evidence that more than 60% of Polish adolescents are characterized as highly active with dominating activities related to walking [19]. It could be that parents perceive that the built environment features, such as neighborhood safety, access to recreation facilities, and crime, limit their child’s physical activity, including active transportation, and this has been found in other studies [20,21,22,23,24]. Lack of pedestrian safety structures, such as crosswalks, might be a significant barrier to active transportation to school in some neighborhoods, which has been previously reported [25]. Safety was described both as the absence of crime and related to features such as lighting with influence of maintenance and renovation [26]. The importance of safety has also been identified to be a more relevant aspect for girls than for boys [27], and many parents do not allow their children to go outside alone due to fear of crime and traffic [28]. As mentioned in ecological models of health behavior [29], we need to understand how people interact with their environments. There is a need to study neighborhood safety in association with PA in adolescents, which is still not well-described and understood. As far as we know, no study on the safety of a neighborhood has been conducted in Poland nor in the Czech Republic. Both these countries are dealing with similar social, cultural, and historical development, and share the same issues on PA. Therefore, the purpose of this study was to examine the associations between the physical activity of Czech and Polish adolescents and their perceptions of the safety of their neighborhood environment.

## 2. Materials and Methods

### 2.1. Participants and Setting

In total, 2001 secondary school students (849 boys and 1152 girls) from the Silesian-Katowice region in Poland (24 schools) and the Moravian regions in the Czech Republic (35 schools) took part in the survey of weekly PA and its environmental correlates (Table 1) during the 2008–2009 and 2013–2014 school years. Moreover, schools interested in deeper analyses of student physical activity agreed to objective PA monitoring. A total of 19 schools (6 from the Czech Republic and 13 from Poland) consented, and 550 selected adolescents (236 boys, 314 girls) participated in a weekly objective monitoring of PA using the YAMAX SW-700 pedometers (Yamax Corporation, Tokyo, Japan). Their results represent the different levels of PA based on perceived environment safety, but no other details are given. All subjects gave their informed consent for inclusion before they participated in the study. The study was conducted in accordance with the Declaration of Helsinki, and the protocol was approved by the Ethics Committee of Faculty of Physical Culture at Palacký University Olomouc (No. MSM6198959221 and No. 37/2013).

### 2.2. Instruments

The Neighborhood Environment Walkability Scale-Abbreviated (NEWS-A) was used to collect data and is a reliable and valid survey of physical-activity-related neighborhood environment construct [22,30]. This questionnaire assesses the perception of neighborhood design features related to physical activity, including residential density, land-use mix (including both indices of proximity and accessibility), street connectivity, infrastructure for walking/cycling, neighborhood aesthetics, traffic hazards, and crime safety. Each of these scale scores was computed as the mean of constituent item responses. Items in the remaining scales were all rated on a 4-point scale from “strongly disagree” to “strongly agree” with scale scores computed as the means of item responses. All subscales have previously shown moderate to good test–retest reliability (*r* = 0.56–0.87) [22]. The questionnaire was filled in by adolescents via the International Database for Research and Educational Support system (www.indares.com). The questions related to neighborhood safety regarded infrastructure for walking/cycling, traffic hazards, and crime safety. Scoring procedures was done in agreement with results of multi-level confirmatory factor analysis [31]. All answers were scored on block-group level (mean of items) and on the scale quartiles of the domain characterized the levels of perceived neighborhood environmental safety. The questions characterized safety from NEWS-A were as follows:

1. There is so much traffic along nearby streets that it makes it difficult or unpleasant to walk in my neighborhood.
2. The speed of traffic on most nearby streets is usually slow (30 mph or less).
3. Most drivers exceed the posted speed limits while driving in my neighborhood.
4. My neighborhood streets are well lit at night.
5. Walkers and bikers on the streets in my neighborhood can be easily seen by people in their homes.
6. There are crosswalks and pedestrian signals to help walkers cross busy streets in my neighborhood.
7. There is a high crime rate in my neighborhood.
8. The crime rate in my neighborhood makes it unsafe to go on walks during the day.
9. The crime rate in my neighborhood makes it unsafe to go on walks at night.

The level of PA was assessed using the International Physical Activity Questionnaire-Long Form (IPAQ-LF) in Polish and Czech adolescents [32] via the www.indares.com. The translation of the questionnaire was carried out in line with recommendations of the European Organisation for Research and Treatment of Cancer (EORTC) Quality of Life Group [33]. The IPAQ-LF questionnaire covers various kinds of PA (job/school-related PA; transportation PA; housework, house maintenance, and caring for family; recreation, sport, and leisure-time PA), various intensities of PA (vigorous, moderate and walking), and time spent sitting. To avoid overestimation of time spent by PA and underestimation of time spent sitting [34] and to maintain a composition of weekly PA that is as objective as possible, the following adjustments to the questionnaire were made: (a) multiplication of MET-minutes of vigorous PA by 6 (instead of original 8); (b) transfer of the estimates of weekly minutes of PA for specific sorts of PA to average daily minutes of PA; (c) capping of the average daily sum of minutes of PA and transportation at 600 min; and (d) capping of the maximum eligible amount of MET-minutes at 20,000. The formula for computation walking MET-min/week was: 3.3 × walking min × walking days. The formula for computation moderate MET-min/week was 4 × moderate intensity activity minutes × moderate intensity activity days. These conditions were not met by 135 participants who were excluded from the final sample. Adolescents were classified as meeting physical activity guidelines if the mean score indicated they were active for at least 60 min per day 5 or more days per week in agreement with moderate-to-vigorous physical activity guidelines [1].

Following the questionnaire survey, Yamax Digiwalker SW-700 (Yamax Corporation, Tokyo, Japan) pedometers were used for objective monitoring of week-long PA in students who were willing to do so (including both school and weekend days). Extreme values of daily step counts were treated in accordance with a generally recognized method [35]. Data were screened for extreme values and data for any single day indicating <1000 steps were removed, and values >30,000 steps on any single day were truncated to 30,000 steps. Equivalent cut points have been used to identify outliers among younger individuals. Moreover, these cut points appear reasonable for our data given that the minimum and maximum daily steps found in a previous studies were identified [36,37]. Those participants who did not provide data for at least 3 school days and 1 weekend day were excluded from further analyses (*n* = 45). Missing days were replaced by values of the closest school or weekend day (77 days were replaced in this manner). The recommendations of a daily step count of 11,000 steps/day were set both for boys and girls, in line with Tudor-Locke et al. [38]. The participants wore the pedometers from the morning (after personal hygiene) for the entire day (except for swimming or bathing).

### 2.3. Data Analysis 

We used descriptive statistics, crossing tables with Pearson’s χ^2^, Kruskal–Wallis ANOVA, repeated measures ANOVA, bilogistic regression, and η^2^, w, and ω^2^ effect size coefficients [39]. The data was analyzed in programs SPSS version 22 (IBM SPSS, Inc., Armonk, NY, USA) and Statistica version 13 (StatSoft, Prague, Czech Republic).

## 3. Results

**Figure 1:**
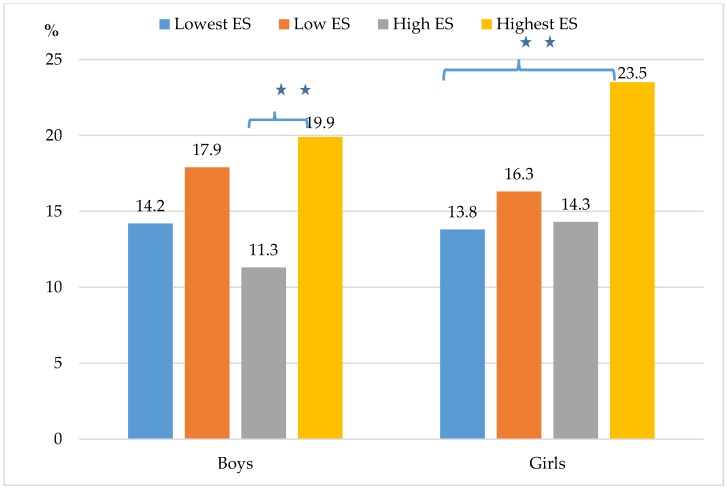
Rates of boys (*n* = 849) and girls (*n* = 1152) meeting the leisure-time walking activities (5 × 60 min walking per week) recommendations by different levels of perceived neighborhood environmental safety (ES) (** = *p* < 0.01).

Boys and girls who perceived their neighborhood environment as the safest met the recommendations for leisure-time walking activities, for at least 60 min per day on at least 5 days per week, at significantly higher rates than those who lacked these perceptions of safety. Boys who perceived their neighborhood environment with the highest safety met the recommendations for leisure-time walking activities significantly more than boys perceiving their neighborhood safety as high (χ^2^ = 7.17; *p* = 0.007; w = 0.092). Girls who perceived their neighborhood environment with the highest safety met the recommendations for leisure-time walking activities significantly more than girls perceiving their neighborhood safety as lowest (χ^2^ = 7.25; *p* = 0.007; w = 0.079). Due to the low levels of effect sizes, we do not consider these differences as logically significant (Figure 1).

We did not find significant differences between boys living in the areas perceived safest and boys living in the areas perceived most dangerous. Similarly, the differences in overall activities indicate that the associations between a safe neighborhood environment and weekly PA did not play an essential role in boys’ activities (Table 2).

However, the differences observed in girls’ transportation and leisure-time walking activities in terms of perceived associations between the environment and walking activities are significant (Table 3).

The perception of a safe neighborhood environment increased odds for meeting the recommendations for leisure-time walking activities in girls (OR = 1.92, 95% CI = 1.189–3.103, *p* = 0.008). Control variables in Model 2 showed neither a significant influence on walking nor a decrease in odds for meeting the recommendation for walking activities in a safe neighborhood environment (OR = 1.80, 95% CI = 1.089–2.96, *p* = 0.022). Our results suggest that the number of girls meeting the recommendation for walking activities was affected mostly by a lack of pedestrian crossings and traffic lights; 20% of girls who perceived the environment as safe met the recommendation, while in the least safe environment the rate was only 12% (*p* = 0.014).

**Figure 2:**
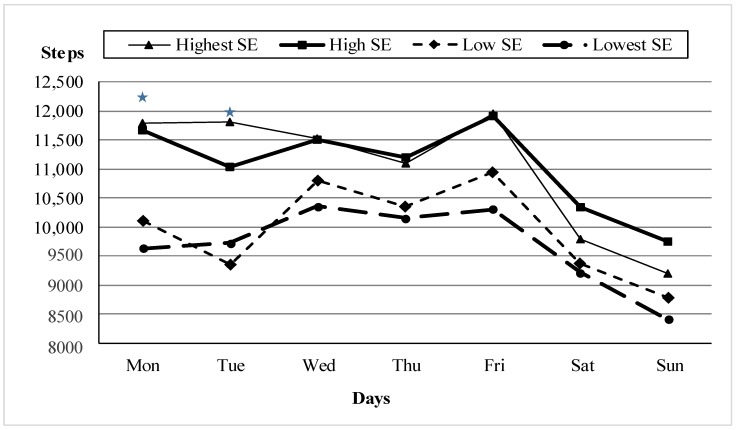
Adolescents’ average daily step count for each day of the week (*n* = 550) according to environmental safety (ES) (* = *p* < 0.05).

Regardless of respondents’ sex, differences in average daily step count in adolescents living in environments with varying levels of self-perceived safety (F (3, 550) = 6.75; *p* ˂ 0.001; ω^2^ = 0.030) corresponded with results obtained by the self-rated estimate of PA. Adolescents from the safest environment achieved 11,024 steps/day on average, while those from the least safe environment achieved only 9686 steps/day (*p* = 0.017). Regarding the specific day of the week, we found significant differences on Mondays (*p* = 0.008) and Tuesdays (*p* = 0.006). The differences were less pronounced on other days of week (Figure 2). Repeated measures ANOVA including the factor of sex (days × sex × safety) did not confirm significant differences (F (18, 550) = 1.05; *p* = 0.394) concerning specific days of the week.

However, we observed significant differences in rates of meeting the recommendation of 11,000 steps/day between boys living in the safest environment and boys living in the least safe environment on Mondays (52.7% vs. 31.0%; *p* = 0.019), Tuesdays (52.7% vs. 32.8%; *p* = 0.032), and Fridays (60.0% vs. 41.4%; *p* = 0.048). In girls, these differences were significant on Mondays (54.2% vs. 25.3%; *p* = 0.001), Tuesdays (54.2% vs. 34.2%; *p* = 0.027), and Wednesdays (50.0% vs. 31.74%; *p* = 0.039). Criminality in the neighborhood environment probably influences rates of meeting the recommendation in boys (48% of boys meet the recommendation in a safe environment and 21% of boys in a dangerous environment) (*p* = 0.026).

Similarly to the self-rated assessment of PA, the rate of girls meeting the 11,000 steps/day recommendation on school days is particularly influenced by a lack of pedestrian crossings and traffic lights (71% of girls met the recommendation in the safest environment, while only 49% of girls in the least safe environment achieved the recommendation (*p* = 0.029). Furthermore, the overall rating of criminality played a role in the level of PA in girls (50% vs. 23%) (*p* = 0.002) as well as in boys (59% vs. 26%) (*p* = 0.001).

## 4. Discussion

The main aim of this study was to examine the associations between physical activity of Czech and Polish adolescents and their perceptions of neighborhood safety. When adolescents perceived their neighborhood environment as safe, they were significantly more likely to meet PA guidelines, and they achieved 11,024 steps/day on average, compared to adolescents living in neighborhoods perceived as unsafe with 9686 steps/day. This indicates that safety is gradually becoming an important issue with regard to assessment of and involvement in PA in the Czech Republic and Poland, which is similar to more developed countries. Previous studies of PA determinants in adolescents have reported that there was some evidence for a correlation between parents’ perceptions of safety and their children’s PA levels [40]; however, the majority of studies have reported non-significant effects [41,42]. This might be influenced by the fact that most of the studies measured overall levels of PA rather than context-specific PA behaviors [42].

As in other studies [37,43], boys had a higher level of total PA than girls. In girls, we observed in particular a significant difference in meeting the PA and walking for transport recommendations (20% in a safer neighborhood compared to 12% in an unsafe one), which applied to the patterns of all types of walking, that is, recreational, for transport, and occupational which hardly differ by sex [44,45]. This finding agreed with a clear indicator found in previous studies that pedestrian safety infrastructure significantly limits active transport to school [25]. It has already been documented that most children who were active travelers were those who lived close to school [46,47,48]. The transportation mode to school might be often chosen by parents who usually report distance as a common barrier to walking or bicycling to school [20,22,24]. The correlates of children’s PA such as traffic speed and volume, and access or proximity from home to recreation facilities or schools, might be a potential safety barrier to being physically active [8,9]. The findings in our study indicate that a safe neighborhood environment could be a predictor for active transport in adolescents and thus could help meet daily PA recommendations [3,24]. Since it has been reported that physical activity levels among adolescents tend to be higher in outdoor rather than indoor spaces [15,49], it is necessary to support safe environments for adolescents during their out-of-school time. Understanding how people interact with their environment is necessary to create environments and policies that make it convenient, attractive, and healthful to enjoy outdoor activities [29]. If adolescents have safe access to a park, they are less likely to be inactive than those without such access [50,51], and these associations are a signal for promoting safe places for healthy activities.

In general, the number of steps/day significantly differed by the day of the week in our sample. Similar findings were observed in adults in the United States [52] where the day-to-day variability in steps/day was consistent with the fluctuations in PA associated with the day of week. A previous study in the Czech Republic also identified the day-to-day differences in the variability of PA [53]; however, it is in contrast to a U.S. study that noted that the number of steps/day did not vary significantly and that the PA behavior of adolescents may be more stable than that of adults [43]. It is possible that with an objective measure of physical activity and the environment, such as actual crime reports, the mediating effects of neighborhood walkability and safety on the education-physical activity relationship might be better elucidated. Studies of this type should become a priority [3].

The findings of this study have implications for school and public health interventions that aim to increase PA in adolescents. The promotion of changes for access to recreation facilities and traffic safety should support efforts that aim to reduce neighborhood barriers and improve safety, which would supposedly also result in an increase of PA in adolescents.

Key strengths of this study include the context-specific measures of PA using the online educational system and the objective measure of PA on a large sample. A key limitation is the cross-sectional design, which restricts the ability to generalize the results. Another limitation could be the use of self-reported measures of PA and the specification of safety in the neighborhood. Further, we did not consider objective measures of the neighborhood environment, which might be slightly different in Czech Republic and in Poland. Finally, in this study, the safety of the environment perceived by parents was not considered, and parents likely influence their child’s PA and behavior.

## 5. Conclusions

In our sample of adolescents, we found significant associations between PA such as walking and the perceived level of neighborhood safety. The associations were stronger for girls than boys. Environment safety was also a strong predictor for daily steps in our sample, especially on school days, but not on weekends. Aspects of the built environment may be potential targets for increasing PA among children. Future research should continue to explore potential differences in behaviors in longitudinal and interventional studies and use more objective measures of PA and environmental determinants.

## Figures and Tables

**Table 1:** Sample characteristics.

Characteristics	*n*	Age (Years)		Weight (kg)		Height (cm)		BMI (kg·m^−2^)	
		*M*	*SD*	*M*	*SD*	*M*	*SD*	*M*	*SD*
**Boys**	849	15.81	0.79	67.66	10.75	176.93	7.85	21.56	2.82
**Girls**	1152	15.89	0.75	57.51	8.27	166.83	6.22	20.64	2.53

BMI—Body Mass Index; *M*—mean; *SD*—standard deviation.

**Table 2:** Weekly physical activity in boys (*n* = 849) by different levels of perceived neighborhood environmental safety (ES).

Physical Activity	Boys				H	*p*	η^2^
	Lowest ES (*n* = 155)	Low ES (*n* = 189)	High ES (*n* = 274)	Highest ES (*n* = 231)			
	Mdn (IQR)	Mdn (IQR)	Mdn (IQR)	Mdn (IQR)			
Transportation-walk (MET-min/week)	660 (1188)	495 (1139)	528 (891)	578 (941)	0.50	0.919	0.001
Leisure Time-walk (MET-min/week)	396 (726)	330 (729)	347 (693)	396 (1089)	2.52	0.472	0.003
Leisure Time-moderate PA (MET-min/week)	120 (720)	0 (480)	200 (480)	160 (540)	2.85	0.415	0.003
Leisure Time-vigorous PA (MET-min/week)	420 (1440)	720 (2160)	540 (1440)	720 (2160)	7.97	0.047	0.009
Leisure Time Total PA (MET-min/week)	1788 (2718)	1920 (2638)	1437 (2124)	1902 (2747)	7.41	0.060	0.009
Total PA (MET-min/week)	8058 (6603)	6522 (6235)	5832 (6381)	6624 (5567)	12.25	0.007	0.014

Note. *n* = number, Mdn = median, IQR = interquartile range, H = Kruskal–Wallis test, *p* = level of significance, η^2^ = effect size coefficient.

**Table 3:** Weekly physical activity in girls (*n* = 1152) by different levels of perceived neighborhood environment safety (ES).

Physical Activity	Girls				H	*p*	η^2^
	Lowest ES (*n* = 225)	Low ES (*n* = 301)	High ES (*n* = 379)	Highest ES (*n* = 247)			
	Mdn (IQR)	Mdn (IQR)	Mdn (IQR)	Mdn (IQR)			
Transportation-walk (MET-min/week)	693 (1089)	693 (1056)	594 (1139)	792 (1502)	8.16	0.043	0.007
Leisure Time-walk (MET-min/week)	396 (924)	396 (693)	396 (660)	528 (990)	10.85	0.013	0.009
Leisure Time-moderate PA (MET-min/week)	0 (360)	0 (240)	0 (360)	0 (240)	2.85	0.415	0.002
Leisure Time-vigorous PA (MET-min/week)	300 (840)	360 (1050)	360 (1080)	360 (1260)	1.97	0.580	0.002
Leisure Time Total PA (MET-min/week)	1200 (1770)	1131 (1614)	1133 (1884)	1476 (2178)	7.36	0.061	0.006
Total PA (MET-min/week)	5082 (5665)	5142 (5553)	5073 (5538)	5835 (5696)	2.10	0.552	0.002

Note. *n* = number, Mdn = median, IQR = interquartile range, H = Kruskal–Wallis test, *p* = level of significance, η^2^ = effect size coefficient.
